# Direct Prediction of Phonon Density of States With Euclidean Neural Networks

**DOI:** 10.1002/advs.202004214

**Published:** 2021-03-16

**Authors:** Zhantao Chen, Nina Andrejevic, Tess Smidt, Zhiwei Ding, Qian Xu, Yen‐Ting Chi, Quynh T. Nguyen, Ahmet Alatas, Jing Kong, Mingda Li

**Affiliations:** ^1^ Quantum Matter Group MIT Cambridge MA 02139 USA; ^2^ Department of Mechanical Engineering MIT Cambridge MA 02139 USA; ^3^ Department of Materials Science and Engineering MIT Cambridge MA 02139 USA; ^4^ Computational Research Division Lawrence Berkeley National Laboratory Berkeley CA 94720 USA; ^5^ Center for Advanced Mathematics for Energy Research Applications Lawrence Berkeley National Laboratory Berkeley CA 94720 USA; ^6^ Department of Physics MIT Cambridge MA 02139 USA; ^7^ Advanced Photon Source Argonne National Laboratory Lemont IL 60439 USA; ^8^ Department of Electrical Engineering and Computer Science MIT Cambridge MA 02139 USA; ^9^ Department of Nuclear Science and Engineering MIT Cambridge MA 02139 USA

**Keywords:** density of states, machine learning, phonons

## Abstract

Machine learning has demonstrated great power in materials design, discovery, and property prediction. However, despite the success of machine learning in predicting discrete properties, challenges remain for continuous property prediction. The challenge is aggravated in crystalline solids due to crystallographic symmetry considerations and data scarcity. Here, the direct prediction of phonon density‐of‐states (DOS) is demonstrated using only atomic species and positions as input. Euclidean neural networks are applied, which by construction are equivariant to 3D rotations, translations, and inversion and thereby capture full crystal symmetry, and achieve high‐quality prediction using a small training set of $\approx 10^{3}$ examples with over 64 atom types. The predictive model reproduces key features of experimental data and even generalizes to materials with unseen elements, and is naturally suited to efficiently predict alloy systems without additional computational cost. The potential of the network is demonstrated by predicting a broad number of high phononic specific heat capacity materials. The work indicates an efficient approach to explore materials' phonon structure, and can further enable rapid screening for high‐performance thermal storage materials and phonon‐mediated superconductors.

## Graphical abstract

A machine‐learning‐based predictive model allows a direct output of phonon density‐of‐states (DOS) by inputting atomic coordinates. The implemented Euclidean neural network preserves crystallographic symmetry and enables high‐quality prediction with small training data. The model allows for fast DOS calculation, reaching ab initio accuracy for 70–80% materials with much lowered computational cost, facilitating materials design especially in alloy systems.

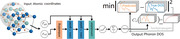

One central objective of materials science is to establish structure‐property relationships; that is, how specific atomic arrangements lead to certain macroscopic functionalities. This question is historically addressed through trial‐and‐error of a combination of structure and property characterization, theory, and calculation. However, recent advances in machine learning (ML) suggest a paradigm shift in how structure‐property relationships can be directly constructed.^[^ ^1^, ^2^ ^]^ To date, ML has seen success in a growing spectrum of materials applications, including materials discovery and design,^[^ ^3^, ^4^, ^5^, ^6^ ^]^ process automation and optimization,^[^ ^7^, ^8^ ^]^ and prediction of materials' mechanical (elastic moduli),^[^ ^9^, ^10^, ^11^, ^12^ ^]^ thermodynamic and thermal transport (formation enthalpy, thermal conductivity, Debye temperature, heat capacity),^[^ ^10^, ^12^, ^13^, ^14^, ^15^, ^16^ ^]^ and electronic (bandgap, superconductivity, topology) properties,^[^ ^11^, ^17^, ^18^, ^19^, ^20^, ^21^, ^22^, ^23^, ^24^ ^]^ and atomistic potentials (potential energy surfaces and force constants).^[^ ^25^, ^26^, ^27^, ^28^, ^29^, ^30^, ^31^ ^]^ Most property prediction studies consider a low‐dimensional output consisting of one or few discrete points. However, the prediction of continuous properties from limited input information remains challenging due to the output complexity and finite data volume. Moreover, for crystalline solids, the crystallographic symmetry poses additional constraints on a generic neural network.

In this work, we build a ML‐based predictive model that directly outputs the phonon density of states (DoS) using atomic structures as input. Phonon DoS is a key determinant of materials' specific heat and vibrational entropy and plays a crucial role in interfacial thermal resistance.^[^ ^32^ ^]^ It is also tightly linked to thermal and electrical transport ^[^ ^33^ ^]^ and superconductivity.^[^ ^34^ ^]^ However, the acquisition of experimental and computed phonon DoS is nontrivial due to limited inelastic scattering facility resources and high computational cost of ab initio calculations for complex materials.^[^ ^33^, ^35^ ^]^ Moreover, the phonon calculations in alloys systems pose significant challenge. Some existing approaches like virtual crystal approximation (VCA) can fail both qualitatively and quantitatively without well‐controlled approximations.^[^ ^36^ ^]^ This calls for an approach that acquires phonon DoS more efficiently, especially for alloy systems. To build such a model, we employ a Euclidean neural network (E(3)NN) which naturally operates on 3D geometry and is equivariant to 3D translations, rotations, and inversion. ^[^ ^37^, ^38^, ^39^, ^40^ ^]^ E(3)NNs preserve all geometric information of the input and eliminate the need for expensive (approximately 500 fold) data augmentation. Additionally, all crystallographic symmetries of input data are preserved by the network.^[^ ^41^ ^]^ In this work, we use E(3)NNs as implemented in the open‐source e3nn repository ^[^ ^40^ ^]^ which merges implementations of Ref. [37] and Ref. [39] and additionally implements inversion symmetry. High‐fidelity phonon DoS predictions are achieved using the density functional perturbation theory (DFPT)‐based phonon database ^[^ ^42^ ^]^ containing phonon DoS data of approximately 1,500 crystalline solids. Our predictive model can capture the main features of phonon DoS, even for crystalline solids with unseen elements. By predicting the phonon DoS in 4,346 new crystal structures, we identify a list of high heat capacity materials, supported by additional DFPT calculations. Our work offers an efficient technique to acquire phonon DoS directly from atomic structure, making it suitable for high throughput materials design with desirable phonon‐related properties.

Crystal structures operated on by the E(3)NN are first converted into a periodic graphs where atoms are nodes N with edges E connecting neighboring atoms within a specified radial cutoff, including periodic images (**Figure** **1**). Each edge $e_{ab}\in E$ stores the radial distance vector between atom a and neighbor $b,{\vec{r}}_{ab}$, up to some radial cutoff $|r_{max}|$, and is used by the convolutional kernels of the E(3)NN. The input node features are scalars that captures its atomic type and mass using one‐hot encoding; for instance, a hydrogen atom is encoded as $x_{H}=\left[m_{H},0,\ldots ,0\right]$. After an initial embedding layer which takes the 118‐length one‐hot mass‐weighted encodings to 64 scalar features, the constructed graph is then passed to the E(3)NN, which iteratively operates on the features with multiple “Convolution and Gated Block” layers as described for the L1Net of ref. [43] (see Supporting Information for more details). After the final layer, which consists of only a convolution, all resulting node features are summed and passed through a final activation (ReLU) and normalization layer to predict the phonon DoS, comprising 51 scalars. The absolute magnitude of the phonon DoS can easily be recovered from the normalized DoS by noticing that ∫g(ω)dω=3N, where N is the number of atoms in the unit cell; thus, we ensure that normalization of the DoS does not compromise meaningful prediction. The E(3)NN weights are optimized by minimizing the mean squared error (MSE) loss function between the DFPT‐computed DoS g and E(3)NN‐predicted $\hat{g}$. The full network structure is provided in the Supporting Information.

**Figure 1:**
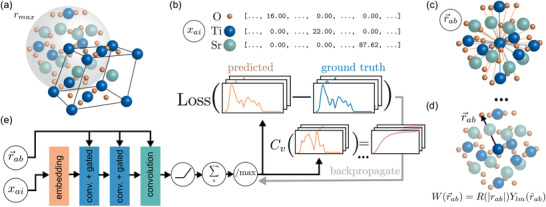
Overview of the E(3)NN architecture for phonon DoS prediction. a) Crystals are converted to periodic graphs by considering all periodic neighbors within a radial cutoff $r_{max}=5$Å. The example of SrTiO${}_{3}$ is shown. b) Atom types are encoded as a mass‐weighted one‐hot encoding. c) Edges join neighboring atoms and store the relative distance vector from the central atom to neighbor. d) The radial distance vectors are used for the continuous convolutional filters $W({\vec{r}}_{ab})$ comprising learned radial functions and spherical harmonics. e) The E(3)NN operates on the node and edge features using convolution and gated nonlinear layers. The result is passed to a final activation, aggregation, and normalization to generate the predicted output. The network weights are trained by minimizing the loss function between the predicted and ground‐truth phonon DoS.

We perform several analyses to evaluate our model given the limited training data. **Figure** **2**a shows that there is no obvious correlation between the MSE and the number of basis atoms within unit cells among training, validation, and test datasets (additional statistics in are available in the Supporting Information). The overall test set error is higher compared to the training set but similar to that of the validation set, suggesting good generalizability. We also present the MSE as a function of different elements (Figure 2b) and observe comparable error levels, indicating balanced prediction. Lastly, we compute the average phonon frequency $\bar{\omega }=\frac{\int d\omega \;g(\omega )\;\omega }{\int d\omega \;g(\omega )}$ for both E(3)NN‐predicted and DFPT ground‐truth spectra, which show excellent agreement on the test set (Figure 2c); specifically, for 70% of the testing samples, the relative error is below 10%. This strongly suggests the capability of our model to predict phonon DoS.

**Figure 2:**
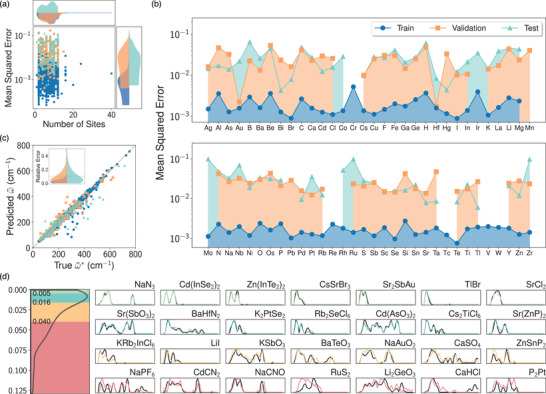
Performance of the Euclidean neural network‐based predictive model. a) Mean squared error versus total number of sites in a unit cell in training (blue), validation (orange), and test (green) sets. b) Average mean squared error of compounds containing each element. c) Comparison between E(3)NN‐predicted average phonon frequency and ground truth. The inset shows the relative error $|\bar{\omega }-{\bar{\omega }}^{*}|/{\bar{\omega }}^{*}$ distribution of the three datasets. d) Randomly selected examples in the test set within each error quartile. (Left) MSE distribution showing that it is heavily peaked in the 1st and 2nd quartiles with lower error.

To visualize the model performance, we plot seven randomly selected examples from the test set in each error quartile in Figure 2d, with rows 1 through 4 corresponding to the 1st quartile with highest agreement through the 4th quartile with lowest agreement, respectively. Additional examples are plotted in the Supporting Information. The predicted DoS in the 1st and 2nd quartiles show excellent agreement with DFPT calculations by reproducing fine features, while the 3rd and 4th quantiles show good or acceptable agreement by capturing main features. For instance, the predicted DoS of NaPF${}_{6}$, CdCN${}_{2}$, and NaCNO capture the energy of acoustic and optical phonon branches well but mispredict the relative amplitudes of certain peaks. Nonetheless, the phonon bandgap, a key quantity to determine phonon‐phonon scattering, can be accurately extracted. Similarly, for KSbO${}_{3}$ and Li${}_{2}$ GeO${}_{3}$, the predictions exhibit broadband DoS distribution, agreeing with DFPT calculations. A large discrepancy can be seen for RuS${}_{2}$, yet the bandwidth agreement is still good although the 0.099 MSE is among the largest errors in the test set (Figure 2a). The good test set performance and generalizability suggest the suitability of our model to predict phonon DoS for a broad range of new materials.

We compare the E(3)NN predictions in six materials with experimental DoS data available from inelastic scattering (**Figure** **3**). Given the disorder and anharmonic effects in a measured sample, disagreement between DFPT calculations and measured data can happen. As a result, lower agreement is expected between experimental and E(3)NN‐predicted DoS since the ground‐truths are based on DFPT calculations.

**Figure 3:**
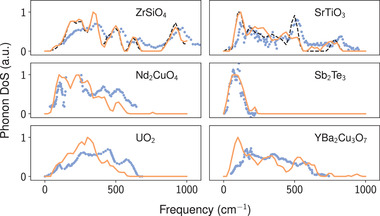
Comparison between E(3)NN model predictions (orange curves) and inelastic scattering DoS data (blue dots). ZrSiO${}_{4}$ was in the training set and SrTiO${}_{3}$ in the test set (black dashed lines denote corresponding DFPT results.^[^ ^42^ ^]^) The remaining examples were absent in all datasets used for training, validation, and testing, and contain two unseen elements, Nd and U. Reproduced with permission.^[^ ^44^, ^45^, ^46^, ^48^, ^49^ ^]^ Copyrights 2008, 1991, 1993, 2014, 2016, American Physical Society;^[^ ^47^ ^]^ Copyright 1981, IOP Publishing,^[^ ^50^ ^]^ Copyright 1997, Elsevier B.V.

Although the E(3)NN‐predicted DoS do not match the fine features of the experimental spectra, several key features (peak positions, gaps, and energy bandwidths) are still well‐predicted and can be valuable in guiding experimental planning, which can serve as useful guidance for planning inelastic neutron and x‐ray scattering measurements, where experimental resources are largely limited to national laboratory facilities.

One of the most important applications of our prediction model may lie in the predictive power in alloy systems, particularly crystalline alloys with substitutional disorders. For example, given a binary alloy with composition $A_{p}B_{1-p}\left(0\leq p\leq 1\right)$, the input alloy encoding vector can take the following form

(1) $$\begin{array}{c} x_{\text{alloy}}=\left[0,\ldots ,pm_{A},\ldots ,\left(1-p\right)m_{B},\ldots ,0\right] \end{array}$$

where the two‐hot encoding $pm_{A}$ and $\left(1-p\right)m_{B}$ are located at the vector indices corresponding the atomic numbers of A and B, respectively, weighted by composition. With this definition of Eq. 1, it can be directly reduced to pure phase one‐hot encoding A (or B) by simply setting p=1 (or p=0), and it can be generalized to more complicated alloys directly. In fact, Equation (1) contains the essence of VCA with both mass average $m_{\text{VCA}}=pm_{A}+\left(1-p\right)m_{B}$ and scattering potential average $V_{\text{VCA}}=pV_{A}+\left(1-p\right)V_{B}$, but combined into one equation: the mass effect is contained in the numerical values of $pm_{A}$ and $\left(1-p\right)m_{B}$, and the potential effect is encoded by turning on the vector indices that correspond to the atomic species A and B. Since the computation of pure phase and alloy differs only by atomic embedding method, the alloy calculation does not generate any additional computational cost.

We demonstrate the power of this approach with the alloy ${\mathrm{Mg}}_{3}{\mathrm{Sb}}_{2(1-p)}{\mathrm{Bi}}_{2p}$ with p∈[0,1]. The model evaluation is done by the aforementioned two‐hot encoding for Sb on top of the structure Mg${}_{3}$Bi${}_{2}$, with simultaneously interpolating the lattice constants in‐between values of two limit structures (namely, Mg${}_{3}$Bi${}_{2}$ for p=1 and Mg${}_{3}$Sb${}_{2}$ for p=0) according to the composition. In this case, both input vectors and the structure are recovered to the pure phase Mg${}_{3}$Sb${}_{2}$ when p=0 and vice versa. We compute the phonon DoS in alloy Mg${}_{3}$Sb${}_{0.5}$Bi${}_{1.5}$ and compare it with VCA calculations, as shown in **Figure** **4**, where the peak positions and magnitudes are both well predicted. The E(3)NN model used to evaluate this alloy system is trained with an additional Mg${}_{3}$Bi${}_{2}$ phonon DoS curated from ^[^ ^51^ ^]^ compared with the model used in the rest of this paper, while Mg${}_{3}$Sb${}_{2}$ has already been included in the original training set.

**Figure 4:**
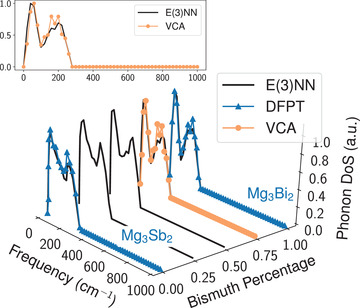
Comparison between E(3)NN model predictions and virtual crystal approximation (VCA) calculations. E(3)NN model can predict phonon DoS for the alloy ${\mathrm{Mg}}_{3}{\mathrm{Sb}}_{2(1-p)}{\mathrm{Bi}}_{2p}$ of continuous p with two‐hot weighted encoding. The triangle‐marked curves indicate DFPT results for Mg${}_{3}$Sb${}_{2}$ and Mg${}_{3}$Bi${}_{2}$.Reproduced with permission.^[^ ^42^ ^]^ Copyright 2018, Springer Nature;^[^ ^51^ ^]^ Copyright 2018, Elsevier B.V. The circle‐marked curve represent VCA calculations for Mg${}_{3}$Sb${}_{0.5}$Bi${}_{1.5}$ (p=0.75). The inset figure shows the front view for E(3)NN and VCA comparison.

We apply the predictive model on 4346 unseen crystal structures without ground‐truth DoS from the Materials Project.^[^ ^52^ ^]^ The data consistency check is performed (Supporting Information), showing a reasonable trend of an elastic spring model. **Figure** **5**a illustrates the average phononic specific heat capacity $C_{V}$ of crystalline solids containing a given element, using the relation ^[^ ^53^ ^]^

(2) $$\begin{array}{c} C_{V}\left(T\right)=\frac{k_{B}}{m_{tot}}\int_{0}^{\infty }{\left(\frac{\hbar \omega }{2k_{B}T}\right)}^{2}{\mathrm{csch}}^{2}\left(\frac{\hbar \omega }{2k_{B}T}\right)g\left(\omega \right)d\omega \end{array}$$

where $m_{tot}$ is the total mass of all N atoms in the unit cell, and the phonon DoS is normalized such that ∫g(ω)dω=3N. Materials containing light elements tend to have high heat capacity, which is reasonable. The distribution of $C_{V}$ evaluated from Equation (2) is shown in Figure 5b, where ≈ 2% of materials show a $C_{V}$ greater than 1000 J(kgK) ${}^{-1}$. The inset shows the average phonon DoS of highest‐$C_{V}$ materials. Materials with higher $C_{V}$ appear to have high spectral weight at higher energies, consistent with expectation. This trend is also noticed by inspecting the scatter plot of phonon DoS along the first two principal components (Figure 5c), where high heat capacity materials appear clustered with respect to the first principal axis. The first principal axis has a broad negative peak extending to high energies; thus, the clustering of high $C_{V}$ materials in the negative first principal direction parallels the shift of their phonon DoS toward higher energies.

**Figure 5:**
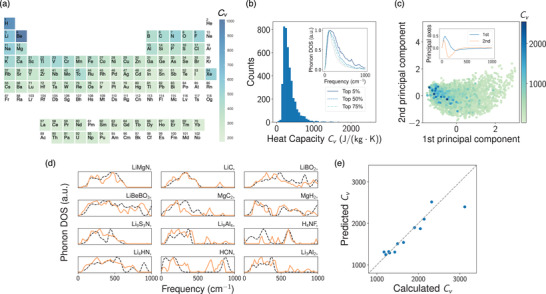
The search for high specific heat capacity ($C_{V}$) materials. a) Periodic table colored by average $C_{V}$ of materials containing each element. b) Histogram showing distribution of $C_{V}$ evaluated from E(3)NN‐predicted phonon DoS. The inset illustrates average phonon DoS of materials with highest $C_{V}$. c) Distribution of predicted phonon DoS along the first two principal components, colored by the E(3)NN‐predicted $C_{V}$ magnitudes. The inset shows first two principal axes in the original frequency basis. d) Comparison between E(3)NN‐predicted and DFPT‐computed phonon DoS, the dashed black curves represent DFPT results. e) 2D histogram comparing specific heat capacities evaluated from E(3)NN‐predicted and DFPT‐calculated phonon DoS.

To validate our model's predictions of high $C_{V}$ materials, we select 12 materials with ultrahigh predicted $C_{V}$ and carry out independent DFPT calculations. Since the maximum frequency of the training data was set to 1000 cm${}^{-1}$, which is sufficient for the majority of materials, the $C_{V}$ evaluated by DFPT was also cut off at 1000 cm${}^{-1}$ for fair comparison. The DoS comparisons between E(3)NN and DFPT in these high‐$C_{V}$ materials are shown in 5d, where satisfactory agreements are achieved in most examples except for H${}_{4}$NF and HCN. The $C_{V}$ at room temperature T=293.15K evaluated from E(3)NN predictions and DFPT calculations are plotted in Figure 5e, showing excellent agreement for most materials. Values of the plotted $C_{V}$ are presented in Table S2, Supporting Information, together with $C_{V}$ computed from full energy ranges for comparison. We attribute the large discrepancy in hydrogen‐ and lithium‐rich materials to the electrostatic effect of hydrogen and lithium bondings beyond mass effect,^[^ ^54^ ^]^ while the current model mainly considers the mass effect.

In this work, we present a machine learning‐based predictive model to directly acquire the high‐dimensional material property of phonon DoS, using only “first‐principles” inputs, namely atomic species and positions. Due to their equivariance, Euclidean neural networks are able to capture the symmetries of the input crystal, making them data‐efficient. A small training set of only 1200 examples is sufficient to generate meaningful predictions, outperforming a well‐trained convolutional neural network even with data augmentation (Supporting Information). One limitation of the current model is identified as samples with elastic strain, potentially related to the fact that we confined output energy range to positive values (Supporting Information, which also includes Refs [56, 57, 58]). Even so, Euclidean neural networks can be applied to predicting broader properties in crystalline solids, where there are often issues of data scarcity. Most importantly, our atomic embedding approach offers an extremely efficient way to compute phonon DoS in alloys, where the alloy prediction has the same computational cost as pure phase by simply changing the 1‐hot embedding to a weighted multi‐hot embedding.

In contrast to ab initio calculations and inelastic scattering that acquire phonon DoS deterministically, a ML model is data‐driven and probabilistic in nature. It is thus impractical to fully rely on a ML‐based predictive model to acquire materials properties without further validation. However, the power of the ML approach goes far beyond obtaining property magnitudes at the individual material level. From a materials design perspective, ML demonstrates extremely high efficiency in rapidly screening candidates with a target property. In our case, the prediction of phonon DoS on the 4346 unseen materials can be done in less than 30 minutes on a single entry‐level GPU. From a property optimization perspective, instead of measuring an individual material with high precision, the ML approach searches outputs high‐performance candidates in a batch, offering more choices. From an experimental perspective, the ML model can be valuable in guiding the experimental planning with limited national facility resources. From an application point‐of‐view, the Dulong‐Petit law poses a grand challenge in searching for promising materials for thermal storage,^[^ ^55^ ^]^ and a highly efficient approach can further support an inverse design from property to desirable structures. In sum, our model provides a promising framework to enable high‐throughput screening and guide experimental planning for materials with exceptional thermal properties. It further sheds light on elucidating the fundamental links between symmetry, structure, and elementary excitations in condensed matter.

## Conflict of Interest

The authors declare no conflict of interest.

## Supporting information

Supporting Information

## Data Availability

The data that support the findings of this study are openly available in [GitHub] at [https://github.com/zhantaochen/phonondos_e3nn].
