# Supplementary material for: Direct Prediction of Phonon Density of States With Euclidean Neural Networks
Source: Adv Sci (Weinh). 2021 Mar 16;8(12):2004214. doi: 10.1002/advs.202004214 (PMC8224435; doi:10.1002/advs.202004214)
Supplement: Supplementary file 1 — Supporting Information [file ADVS-8-2004214-s001.pdf]

© 2021 Wiley-VCH GmbH

ADVANCED SCIENCE

Open Access

Supporting Information

for *Adv. Sci.,* DOI: 10.1002/advs.202004214

Direct Prediction of Phonon Density of States With Euclidean Neural Networks

*Zhantao Chen, Nina Andrejevic, Tess Smidt, Zhiwei Ding, Qian Xu, Yen-Ting Chi, Quynh T. Nguyen, Ahmet Alatas, Jing Kong, and Mingda Li**

Supplementary Material

GENERAL VALIDITY OF PREDICTIONS ON UNSEEN MATERIALS

We apply the predictive model on 4,346 unseen crystal structures from the Materials Project [52] with atomic site number $N \leq 13$ in each unit cell (consistent with most materials presented in [42]). As a check, we plot the average phonon frequency $\bar{\omega}$ against the average atomic mass $\bar{m} = (\frac{1}{N}\sum_{i=1}^{N}\sqrt{m_i})^2$ in the unit cell (Figure S1a). The predicted data fit well to a hyperbolic curve $\bar{\omega} = C\bar{m}^{-1/2}$, where the constant C is a measure of the crystal rigidity. The reasonable distribution of rigidity supports the physical validity of our model for new materials. Moreover, we characterize the non-uniformity of atomic masses in each material by computing the ratio of the minimum mass m_{min} in a crystal to $\bar{m}$, where high $m_{\mathrm{min}}/\bar{m}$ tends to aggregate at lower $\bar{\omega}$ and higher $\bar{m}$ (Figure S1b), which agrees with the features in [42].

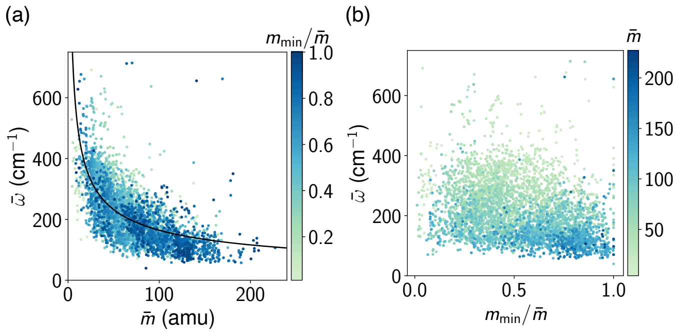

Figure S1. Evaluation of model predictions on unseen crystal structures. (a) Average frequency $\bar{\omega}$ versus average atomic mass $\bar{m}$. The black solid line represents the least squared fit to the hyperbolic relation $\bar{\omega} \sim \bar{m}^{-1/2}$. (b) The average frequency versus the ratio $m_{\mathrm{min}}/\bar{m}$ characterizing atomic mass non-uniformity.

EUCLIDEAN NEURAL NETWORK IN DETAIL

As discussed in the main text, crystals are represented to the network as periodic graphs where relative distance vectors are edge attributes and atom features are stored at each node as shown in Figure 1. Crystal structures are converted into graphs using the `e3nn DataPeriodicNeighbors` class which uses the `pymatgen Structure` class to find atom neighbors while accounting for periodic boundary conditions [56]. For each atom, all atoms around a given atom within a given cutoff radius (including the central atom) are considered "neighbors" and are used in the convolution operation. The features of each atom in the unit cell is stored in x_{ai} where a is the atom index and i is a flattened representation index with features given by a representation list `Rs = [(N, 0, 1)]` which denotes `N` scalars. Representation lists in `e3nn` denote irreducible representations (irreps) of the group of 3D rotations and inversion, $O(3)$, and are lists of triples `(m,` L`, p)` denoting the number of copies or multiplicity `m` of features with rotation degree L and parity `p` (1 for even parity, -1 for odd parity, and 0 for irreps of the group of just 3D rotations $SO(3)$). An irrep of rotation degree L has $2L+1$ components. By flattening the representations into one index and using representation lists, we can efficient store and operate on geometric tensor quantities expressed in the irrep basis.

We employ the modified one-hot encoding for element types with atomic mass as magnitudes, namely $V_{vc}^{(0)}|_{c=Z_v} = m_v$ for site v with atomic number Z_v and mass m_v (note the subscript $m \equiv 1$ is omitted for order $l=0$). For example,

$$\text{H:} \quad [1,0,\ldots,0,0], \qquad \text{He:} \quad [0,4,\ldots,0,0], \qquad \ldots, \qquad \text{Og:} \quad [0,0,\ldots,0,294]. \tag{S1}$$

The encoded crystal graph is then passed into a E(3)NN as illustrated in Figure S2. The architecture of the Euclidean neural networks used in this work is similar to that of a graph convolutional neural networks. To achieve Euclidean symmetry equivariance: 1) E(3)NN convolutional filters are functions of the radial distance vector between two points and composed of learned radial functions and spherical harmonics $W(\vec{r}) = R(|r|)Y_{lm}(\hat{r})$. 2) As a consequence of this filter choice, all inputs, intermediate data, and outputs are geometric tensors. 3) Therefore, all scalar operations (e.g.

addition and multiplication) in the network must be replaced with general geometric tensor algebra. 4) Additionally, nonlinearities applied to geometric tensor data must also be replaced with equivariant equivalents. A feature that emerges from equivariance is that the symmetry of the outputs of Euclidean neural networks are guaranteed to have equal or higher symmetry than the inputs, which means that these networks are guaranteed to respect the space group symmetries (which are a subgroup of Euclidean symmetry) of input crystal geometry [41].

To articulate network operations, we will use Einstein summation notation where repeated indices are implicitly summed over. A single layer of our network operates on input x_{ai} and relative distance vectors of graph edges $\vec{r}_{ab}$,

$$x_{ai}^{(q+1)} = \sigma(x_{bj}^{(q)} \otimes K_k(\vec{r}_{ab})) \tag{S2}$$

where σ is an equivariant nonlinearity and $\otimes$ signifies a tensor product where representation indices of inputs and filter are contracted using Clebsch-Gordan coefficients. We used a "gated" rotation equivariant nonlinearity, `GatedBlockParity` as implemented in `e3nn`, which was first introduced in Ref. [39] and is extended in `e3nn` to handle parity (inversion). K is the convolutional kernel which is composed of learned radial functions and spherical harmonics and Clebsch-Gordan coefficients are included in the kernel to yield the traditional channel out and in indices.

$$K_{ij}(\vec{r}_{ab}) = K_{abij} = R_w(r_{ab})Y_k(\hat{r}_{ab})C_{ijk}\delta_{w,k\in\text{irrep}(w)} \tag{S3}$$

where $\delta_{w,k\in\text{irrep}(w)}$ denotes that radial functions are shared for all components of a given irrep, e.g. the 5 components of a $L = 2$ irrep share the same radial function, and C_{ijk} are the Clebsch-Gordan coeffcients.

In tensor notation, a convolutional operation is written as

$$\text{Conv}(x_{bi}, \vec{r}_{ab}) := x_{bj}K_{ij}(\vec{r}_{ab}) = y_{ai} \tag{S4}$$

We use convolutional filters up to $L \leq 1$ and rotation order for intermediate features of $L \leq 1$.

The radial functions are dense (fully-connected) neural networks acting on a finite radial basis. For example, a two layer radial function would be expressed as

$$R(|r_{ab}|) = W_{kh}\sigma(W_{hq}B_q(|r_{ab}|)) \tag{S5}$$

where B_q are the set of radial basis functions; in this work, we use a finite set of Gaussian radial basis functions.

The first two convolutional layers generate $L = \{0, 1\}$ atomic features and additional scalars to be used by following gated blocks for nonlinearizing $L = 1$ pseudovectors [43]. The final convolution operation yields atomic features of order $L = 0$ on each atom. Finally, states at all sites within the unit cell are aggregated into a one-dimensional array $\sum_{a\in N} x_{ia}^{(q)}$. We then apply a ReLU activation and normalize (by dividing by the maximum intensity) to predict the phonon density of states (DoS), which is simply an array of 51 scalars.

DATA PRE-PROCESSING AND PREPARATION

The phonon DoS dataset with 1,521 crystalline solids calculated from density functional perturbation theory (DFPT) by Petretto et al. [42] is used for training and testing a predictive model in this study. We randomly split the entire dataset into training (80%), validation (10%), and test (10%) sets. We manually curated 3 additional experimental phonon DoS from [57], adding the Cu and Ag examples to the training set and Au to the test set, in order to provide the network examples of single-element compounds. The resulting training set had 1220 samples, and each of validation and test sets had 152 samples.

The DFPT-calculated phonon DoS data has high energy resolution, requiring a large number of parameters in the neural network to fit the output dimension. Given limited training data, it is challenging to train a predictive model with too many trainable weights. To ensure a balanced output dimension and resolution while retaining the main features of the phonon DoS, we interpolated the smoothed spectrum in the energy range $0 \leq \omega \leq 1000\,\text{cm}^{-1}$ to 51 points. The number 51 is well chosen in the sense is that it matches the scattering instrumental resolution. The sampled data points correspond to an energy segmentation of $\sim 2.4\,\text{meV}$ ($20\,\text{cm}^{-1}$), which is roughly the same for typical inelastic scattering measurements with energy resolution $\sim 2.0\,\text{meV}$, below which the finer features are not distinguishable by any existing technique. For instance, the state-of-the-art inelastic scattering at NSLS-II has $\sim 2.0\,\text{meV}$ resolution. It should also be noticed that phonon DoS of some calculated materials in the dataset has negative frequencies components. According to [42], those materials are not accompanied with thermodynamic properties and it is therefore found that around 1/6 of the total data has imaginary modes. However, since our model is confined to

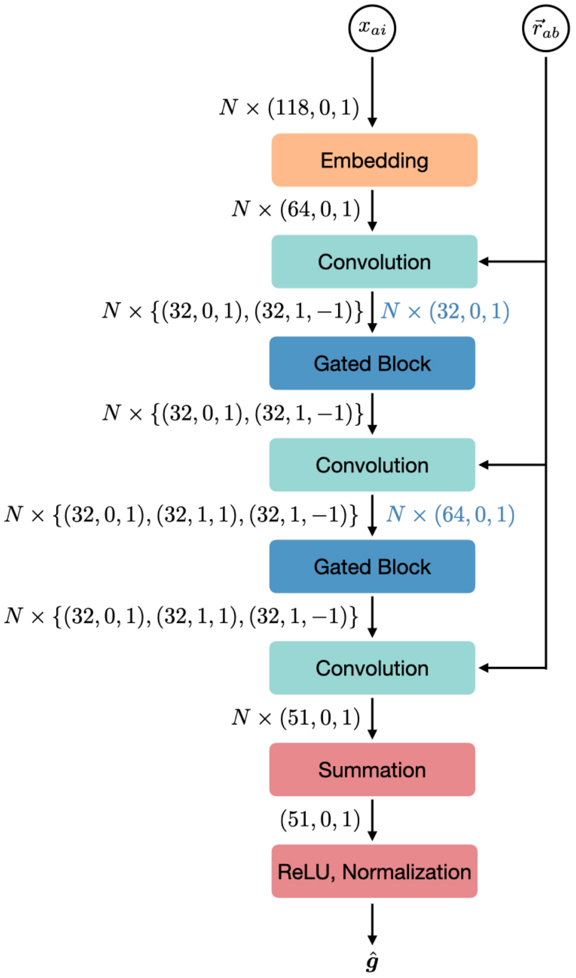

Figure S2. Architecture of the Euclidean neural network.

the energy range $0 \leq \omega \leq 1000\,\text{cm}^{-1}$ and is intrinsically unaware of the truncated information at negative frequencies. This could partially explain the poor behavior of our model in predicting strained samples as it is not trained to differentiate stable and unstable structures.

In particular, smoothing of phonon DoS is achieved by applying a Savitzky-Golay filter of window length 101 and polynomial order 3, where filter parameters are determined to best represent raw phonon DoS: getting rid of small fluctuations while retaining the main profiles. Representative raw and smoothed phonon DoS curves are shown in Figure S3.

HYPERPARAMETER OPTIMIZATION AND NEURAL NETWORK TRAINING

We randomly selected a set of initial hyperparameters within the ranges listed in Table S1 as a starting point for tuning. In addition to the listed parameters, we fixed the maximum radius r_{max} to 5Å, which we found to adequately balance the number of edges created per vertex with the memory requirements to build the corresponding graph. The selected value is also similar to the maximum bond length considered in the CGCNN [11]. We then systematically tested hyperparameters within the specified ranges until an optimal set balancing validation loss and memory limitations was found. The optimal set of hyperparameters, listed in the last column of Table S1, was then used to train the final predictive model.

The neural network is trained with a Quadro RTX 6000 GPU with 24 GB of RAM. We stop updating weights when the validation loss stagnates and tends to rise to reduce over fitting; the loss history can be found in Figure S4.

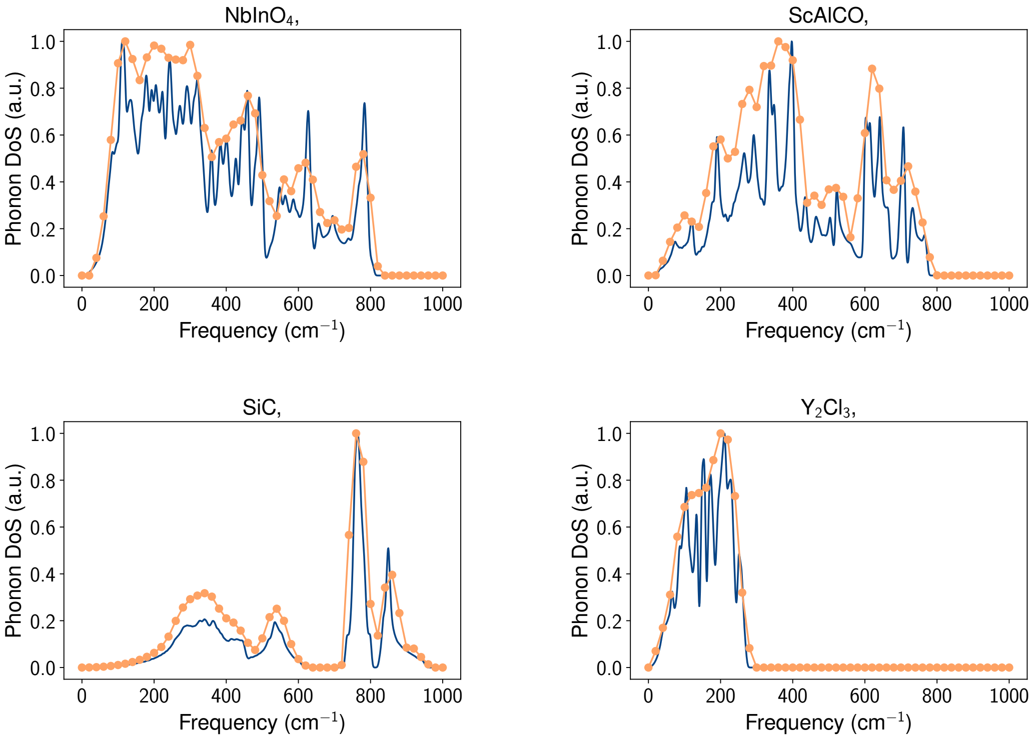

Figure S3. Original and filtered phonon DoS.

Table S1. Hyperparameter searching range and optimization process

Hyperparameter	Range	Initial Selection	Final Selection
Multiplicity of irreducible representation $\mathtt{m}$[a]	16, 32, 48, 64[b]	64	32
Number of pointwise convolution layer	1, 2, 3, 4	3	2
Number of basis for radial filters	5, 10, 15, 20	10	10
Length of embedding feature vector	16, 32, 64, 128, 160	128	64
AdamW optimizer learning rate[c]	$5e^{-4}$, $1e^{-3}$, $5e^{-3}$, $1e^{-2}$	$1e^{-3}$	$5e^{-3} \times 0.96^k$
AdamW optimizer weight decay coefficient	$1e^{-3}$, $1e^{-2}$, $5e^{-2}$, $1e^{-1}$	$1e^{-2}$	$5e^{-2}$

[a] For outputs of first two convolutional layers only.
[b] Out of memory is encountered at value 72.
[c] We observe the lowest validation loss at a learning rate of $5e^{-3}$ among all values tested, followed by oscillations, and thus adopt the exponentially decaying learning rate with k being the epoch number.

MODEL LIMITATIONS ON STRAINED SAMPLES

The proposed machine learning approach has demonstrated success in unseen elements and alloys, but faces one limitation on strained sample at this moment. In Figure S5, we compare the neural network predictions with DFPT calculations for $SrTiO_3$ at three levels of strains. In both cases, the investigated supercells can be evaluated within seconds by a trained E(3)NN neural network. As we can see that the DFPT-computed results are more sensitive than those from E(3)NN, which is attributed to a lack of knowledge of equilibrium bond lengths to be used for the model training and the limited coverage of training data, for example, confined energy range $0 \leq \omega \leq 1000\,\mathrm{cm}^{-1}$ without consideration of imaginary modes. However, better performances are still feasible if more relevant strain-dependent training data can be provided.

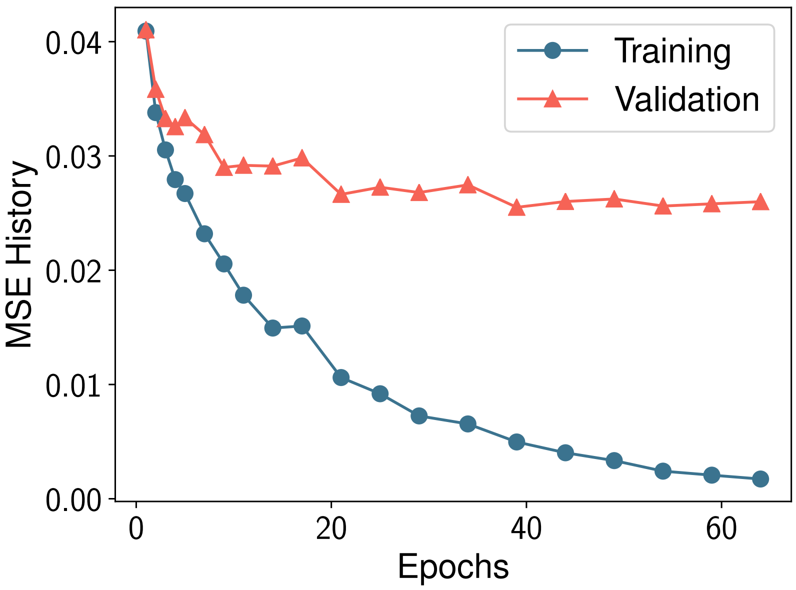

Figure S4. The mean squared error versus epoch number.

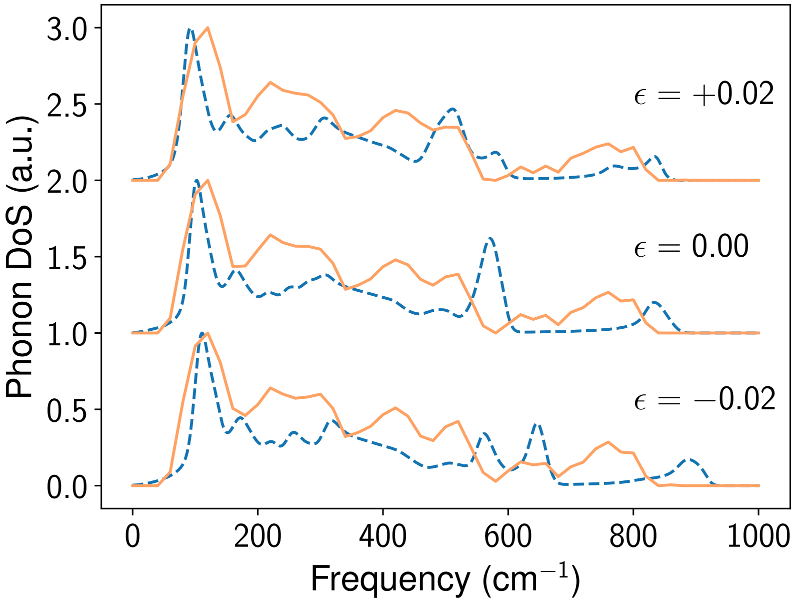

Figure S5. Phonon DoS of $SrTiO_3$ with biaxial strain, including compressional (upper), unstrained (middle), and tensile (lower). The blue-dashed lines and orange lines represent DFPT and E(3)NN predictions, respectively.

TRAINING WITH IMAGINARY PHONON MODE

Considering the fact that in the dataset [42] around 1/6 materials have relatively large negative frequencies in their calculated phonon DoS, indicating a possible poor convergence in the DFPT calculation. We plot the average phonon DoS in Figure S6. The majority of calculated materials have insignificant imaginary modes, with a mean phonon frequency of -6.11 cm^{-1} and a STD of 16.91 cm^{-1} (computed from uninterpolated original data in [42]), computed with the same equation $\bar{\omega} = \frac{\int \mathrm{d}\omega\, g(\omega)\, \omega}{\int \mathrm{d}\omega\, g(\omega)}$ used in Figure 2. It is noticed that most negative frequency phonon modes are located above $-200\,\mathrm{cm}^{-1}$. In order to evaluate the influences of including imaginary phonon modes, we expand the frequency range to $-200 \leq \omega \leq 1000\,\mathrm{cm}^{-1}$ for our model, with same energy interval $20\,\mathrm{cm}^{-1}$ between sampled points such that the final output is an array of length 61, and keep all other hyperparameters unchanged.

In Figure S7, we visualize the losses of the model trained with imaginary phonon modes, where very similar loss distributions are obtained compared with the model trained without imaginary modes in the main article (Figure 2). We further plot true (DFPT) and predicted phonon DoS for materials from training, validation, and test sets in Figure S8, S9, and S10. The example materials in each figure are ordered by the mean magnitude of phonon DoS at negative frequencies. We find that for many materials in the test set, the predictions made in positive energy range are significantly better than in negative frequency ranges, such as Rb_2TlInF_6, $RbHgF_3$, Cs_2As_3, etc. Similar trends can also be observed for the other two data sets. On one hand, the poor performance on the negative frequencies could come from much less informative training samples. On the other hand, the performance on the positive frequencies of our model seems not to be affected significantly by those information.

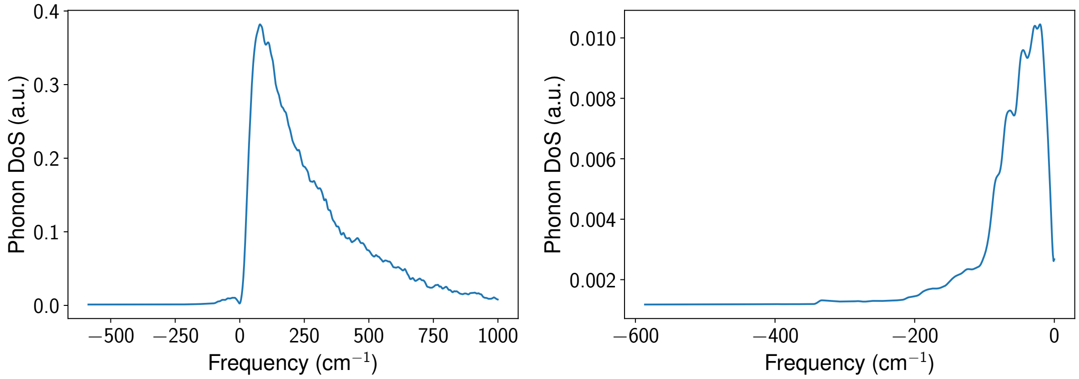

Figure S6. Mean phonon DoS averaged from all 1,521 examples in [42].

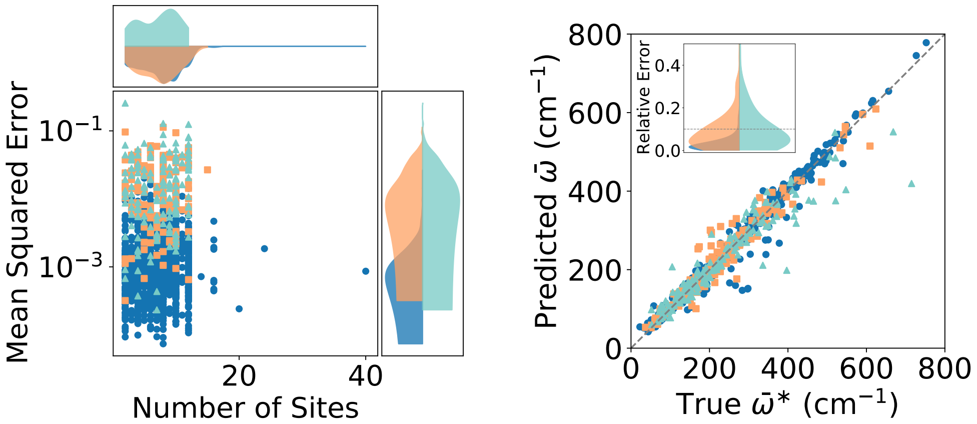

Figure S7. Loss visualization for the model trained with imaginary phonon modes.

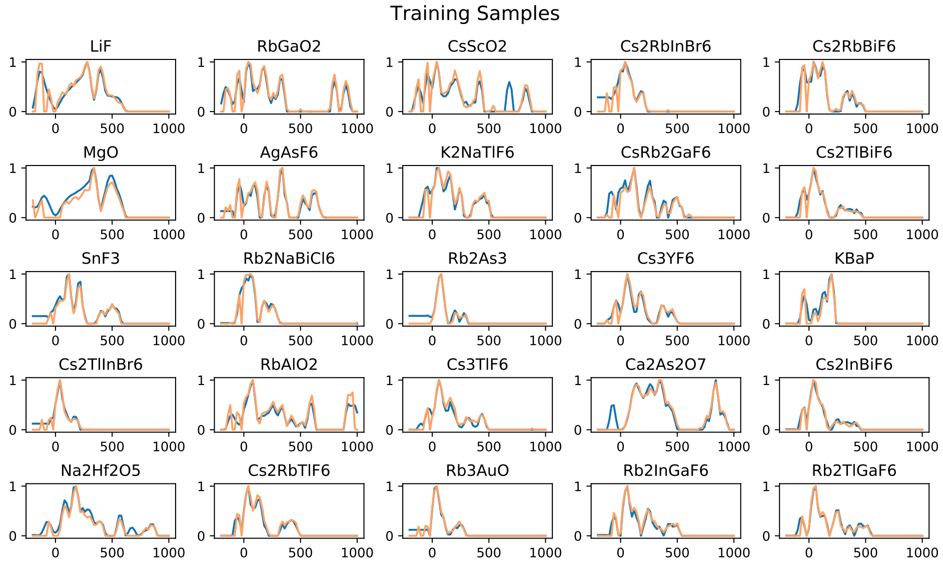

Figure S8. Predicted phonon DoS within training set. Blue and orange curves represent true (DFPT) and predicted phonon DoS, respectively.

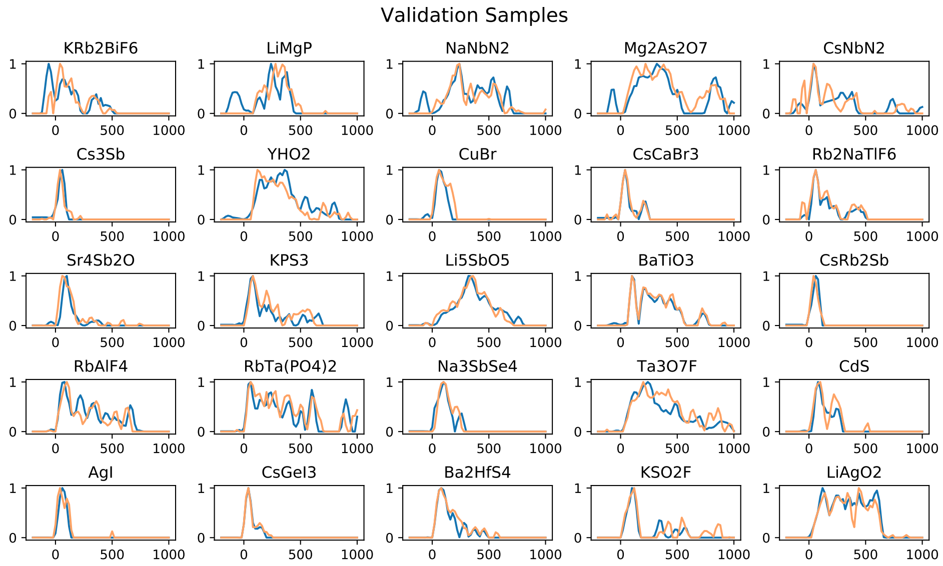

Figure S9. Predicted phonon DoS within validation set. Blue and orange curves represent true (DFPT) and predicted phonon DoS, respectively.

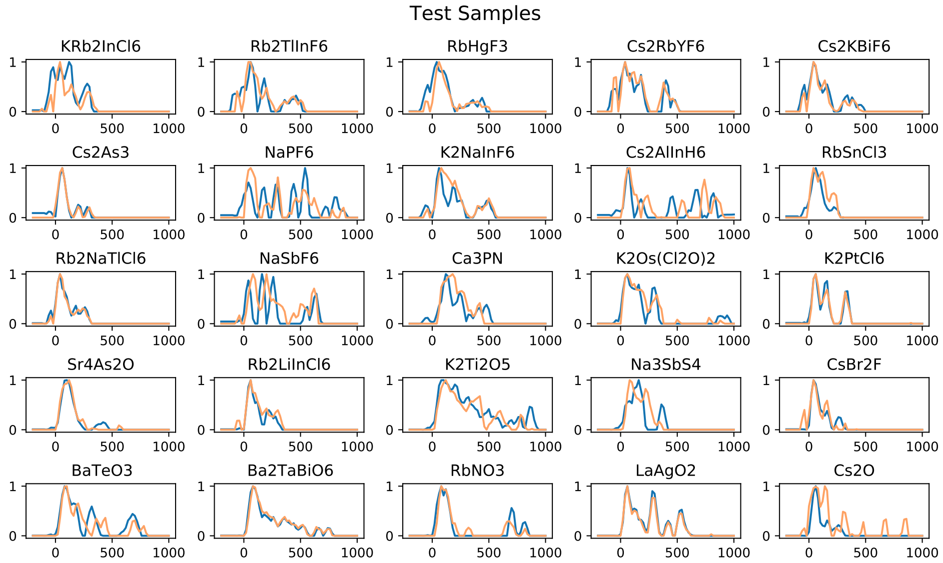

Figure S10. Predicted phonon DoS within test set. Blue and orange curves represent true (DFPT) and predicted phonon DoS, respectively.

MORE PREDICTED PHONON DOS IN TEST SET

We present 100 predicted phonon DoS from the test set on opt of those appeared in the main text in Figure S11. The samples are sorted from top to bottom in order of increasing mean squared error (MSE). As discussed in the main text, our model performs better in predicting energy ranges than exact amplitudes. While near-perfect predictions were achieved for examples in the first few rows, the principal peaks and gaps of the phonon DoS were well-reproduced for many materials located at bottom rows, such as $MgSnAs_2$, LiBeP, $InGaO_3$, and $NaSbF_6$.

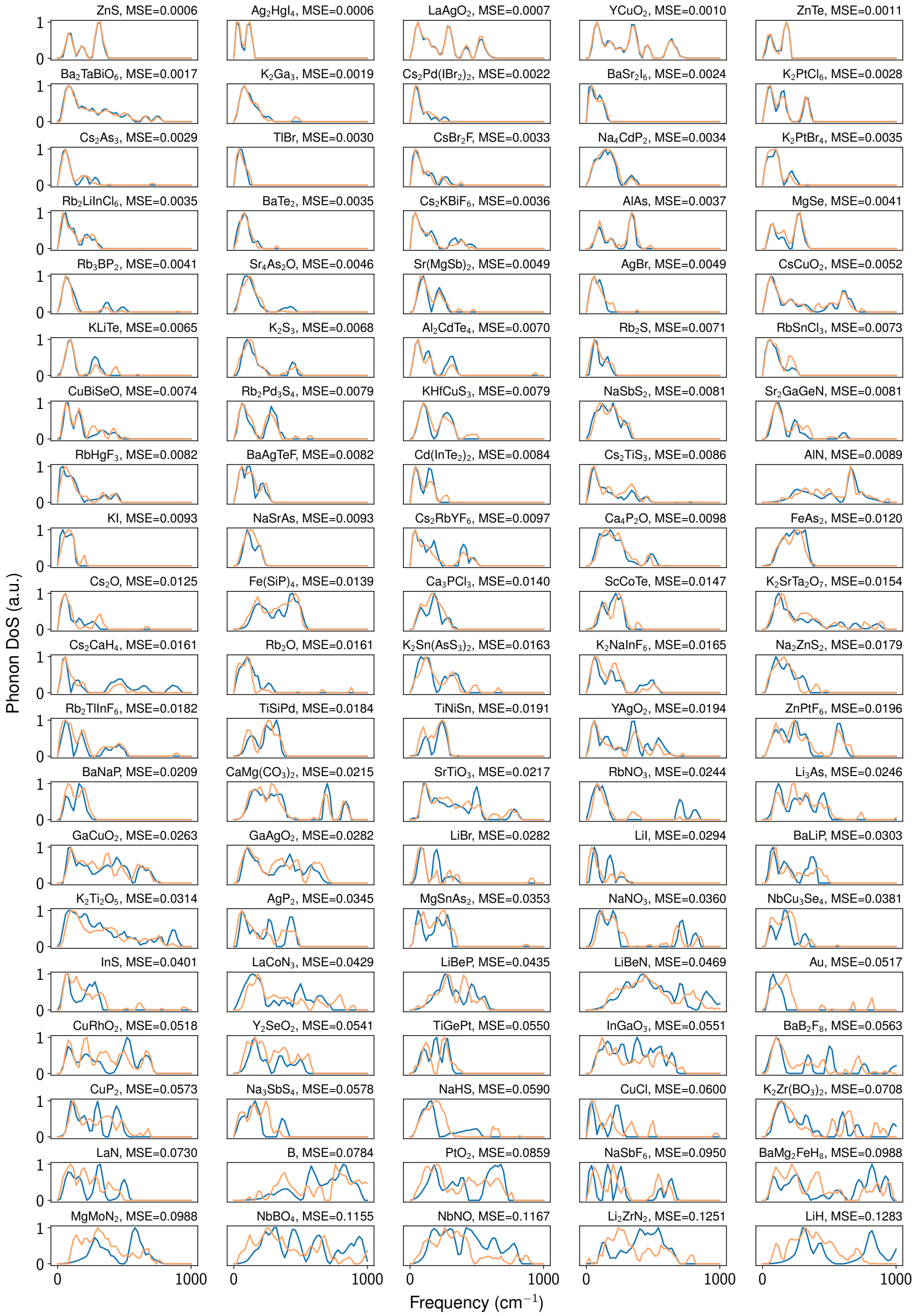

Figure S11. Predicted phonon DoS of randomly-selected examples from the test set, sorted from top to bottom in order of increasing MSE.

ELEMENT REPRESENTATION

In Figure S12, we illustrate the number of occurrences of each element in the training, validation, and test sets. When compared to Figure 2b in the main text, it is found that elements corresponding to high MSE in Figure 2b of the main text tend to appear less frequently than those with lower errors, such as La, Ir, Mo, Rh, and Ru. A more direct comparison is made in Figure S13.

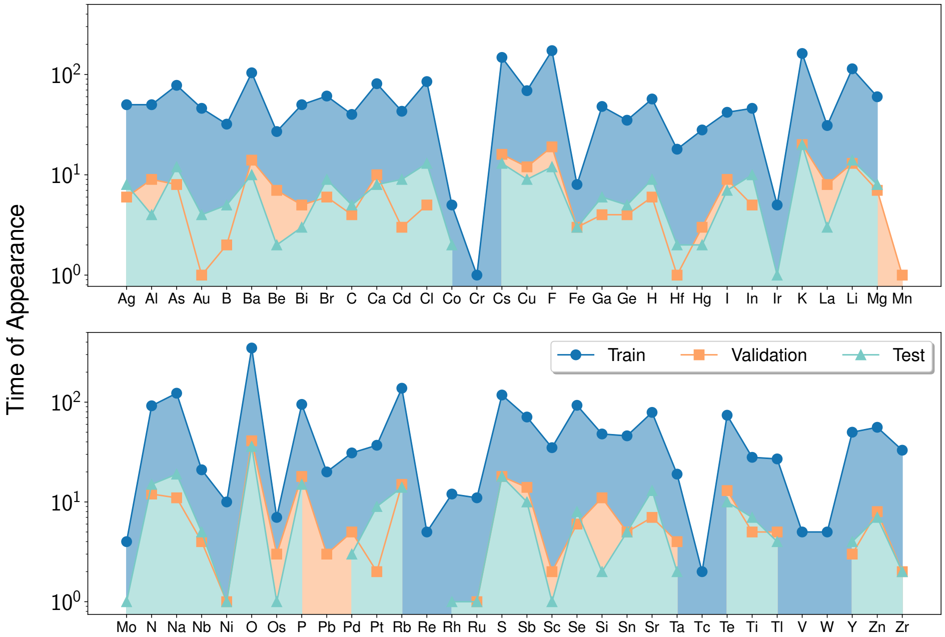

Figure S12. Element representations in training, validation, and test sets.

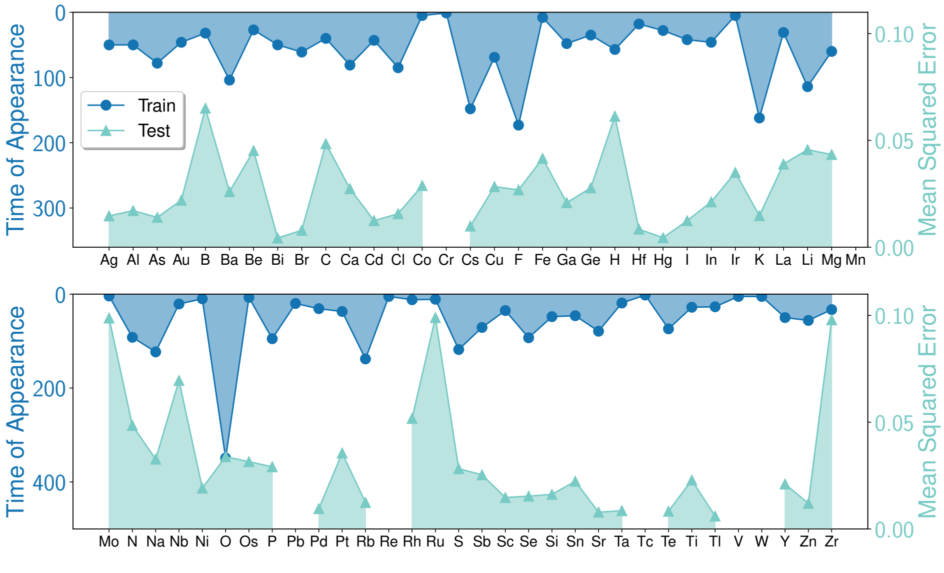

Figure S13. Element representation in training set versus MSE in test set. The inset shows a scatter plot of the relationship.

ADDITIONAL PHONON DOS PREDICTIONS OF MATERIALS WITH HIGHEST SPECIFIC HEAT CAPACITY

We present E(3)NN-predicted materials (selected from 4,346 crystal structures without ground-truth DoS from the Materials Project [52]) with the top 100 highest specific heat capacities in Figure S14.

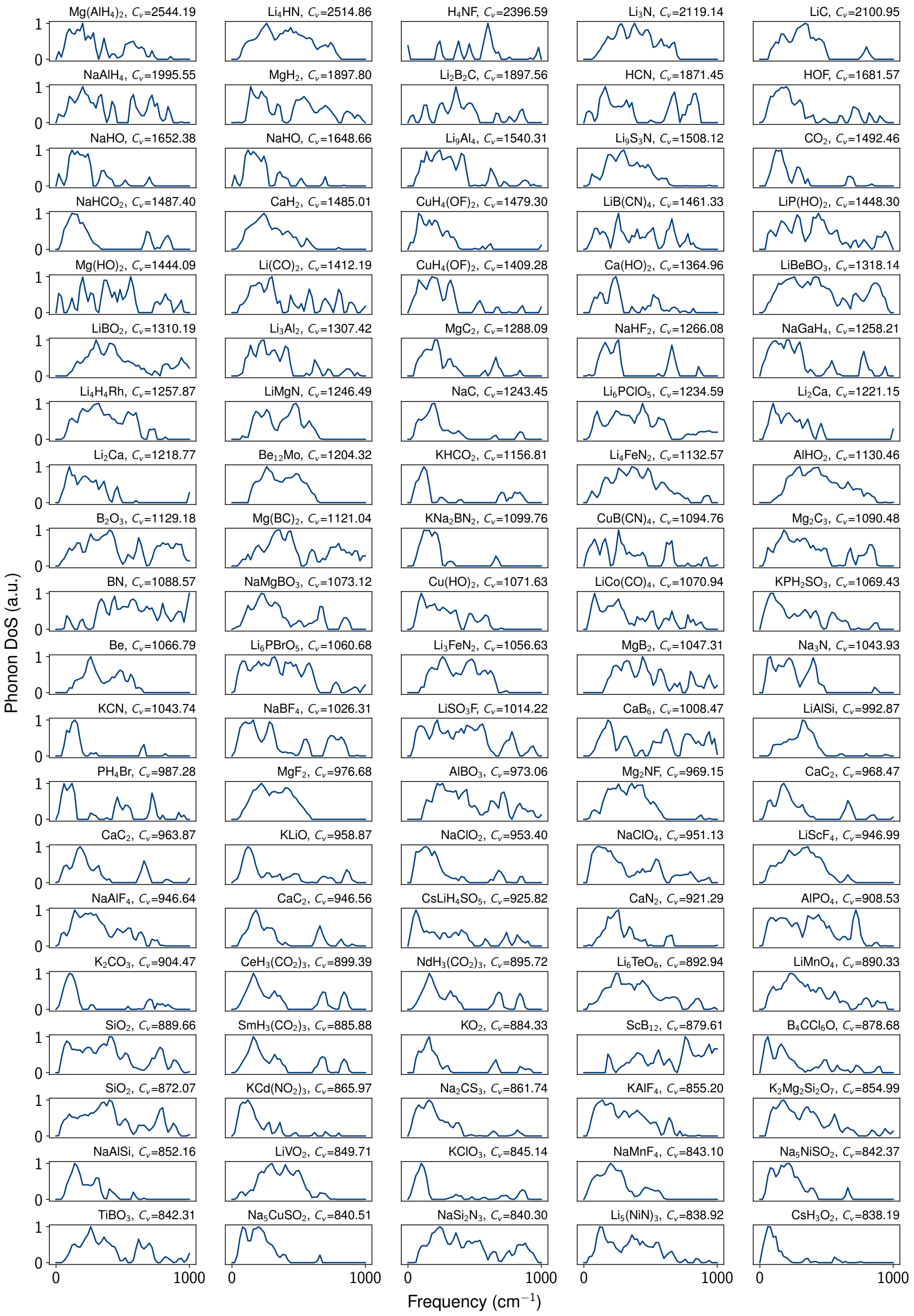

Figure S14. Top 100 high specific heat capacity materials, C_V in unit J/(kg · K).

SPECIFIC HEAT CAPACITY CALCULATED FROM FULL ENERGY RANGES

Due to considerations about neural network size and spatial resolution of the predicted phonon DoS, our model is trained with DFPT-calculated phonon DoS in the energy range $[0, 1000]\,\mathrm{cm}^{-1}$. When evaluating new materials, the E(3)NN-predicted phonon DoS is also confined within this domain. Therefore the specific heat capacities are evaluated from potentially incomplete phonon DoS.

Here we present the specific heat capacities evaluated from the confined and complete phonon DoS in Table S2. It is found that most materials have a slightly different C_V, and 9 out of 12 points are still in good agreement. On the other hand, more dramatic discrepancies in the other 3 materials, MgH_2, H_4NF, and HCN, are partially induced by significant contributions to the DoS at higher energy ranges outside those considered during training. The two-dimensional histogram and corresponding phonon DoS for the three materials are shown in Figure S15. A potential remedy for this issue is to train our model with phonon DoS including higher energy ranges which may lose the energy resolution and is not necessary of majority of materials. The good agreement between E(3)NN-predictions and DFPT ground-truth spectra achieved in the energy range chosen for this work sufficiently demonstrates the potential for generalizability of our model.

Table S2. Comparisons of E(3)NN predicted and DFPT calculated specific heat capacities (in unit J/(kg · K)). Left: C_V is computed from energy range $0 \le \omega \le 1000\,\mathrm{cm}^{-1}$. Right: C_V is computed from full energy range.

Material	$C_V^{E(3)NN}$	C_V^{DFPT}	Material	$C_V^{E(3)NN}$	C_V^{DFPT}
Li_4HN	2514.9	2320.2	Li_9S_3N	1508.1	1498.3
H_4NF	2396.6	3117.4	$LiBeBO_3$	1318.1	1286.1
LiC	2101.0	2138.8	$LiBO_2$	1310.2	1172.9
MgH_2	1897.8	1908.5	Li_3Al_2	1307.4	1431.2
HCN	1871.5	2047.2	MgC_2	1288.1	1307.8
Li_9Al_4	1540.3	1642.1	LiMgN	1246.5	1216.2

Material	$C_V^{E(3)NN}$	C_V^{DFPT}	Material	$C_V^{E(3)NN}$	C_V^{DFPT}
Li_4HN	2514.86	2320.06	Li_9S_3N	1508.12	1498.39
H_4NF	2396.59	1578.65	$LiBeBO_3$	1318.14	1134.99
LiC	2100.95	1959.36	$LiBO_2$	1310.19	1087.88
MgH_2	1897.80	1324.31	Li_3Al_2	1307.42	1431.15
HCN	1871.45	1561.42	MgC_2	1288.09	1152.05
Li_9Al_4	1540.31	1642.07	LiMgN	1246.49	1216.17

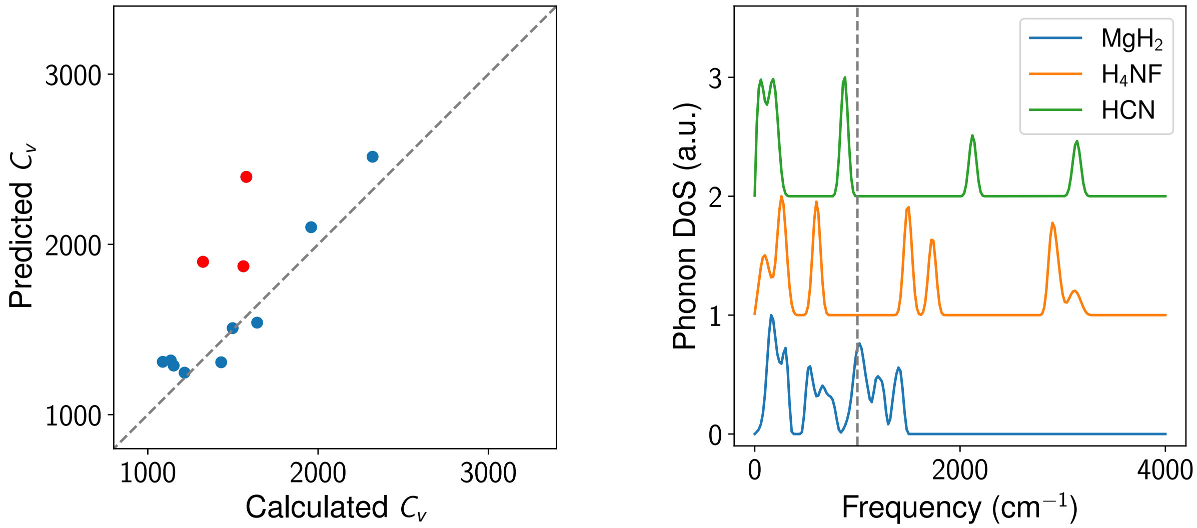

Figure S15. Left: Two-dimensional histogram comparing between specific heat capacities evaluated from E(3)NN-predicted and complete DFPT-calculated phonon DoS. Right: Full phonon DoS of three materials that display largest discrepancies.

Table S3. Architectures of the convolutional neural network.

Layer	Input shape	Output shape
3D conv. layer, kernel size=5, strides=2, channel num.=1<br>activation=ReLU	(32,32,32,2)	(16,16,16,1)
3D conv. layer, kernel size=3, strides=2, channel num.=2<br>activation=ReLU	(16,16,16,1)	(8,8,8,2)
3D conv. layer, kernel size=3, strides=2, channel num.=4<br>activation=ReLU	(8,8,8,2)	(4,4,4,4)
3D conv. layer, kernel size=3, strides=2, channel num.=8<br>activation=ReLU	(4,4,4,4)	(2,2,2,8)
Flatten	(2,2,2,8)	64
Fully connected, activation=ReLU	64	64
Fully connected, activation=ReLU	64	51

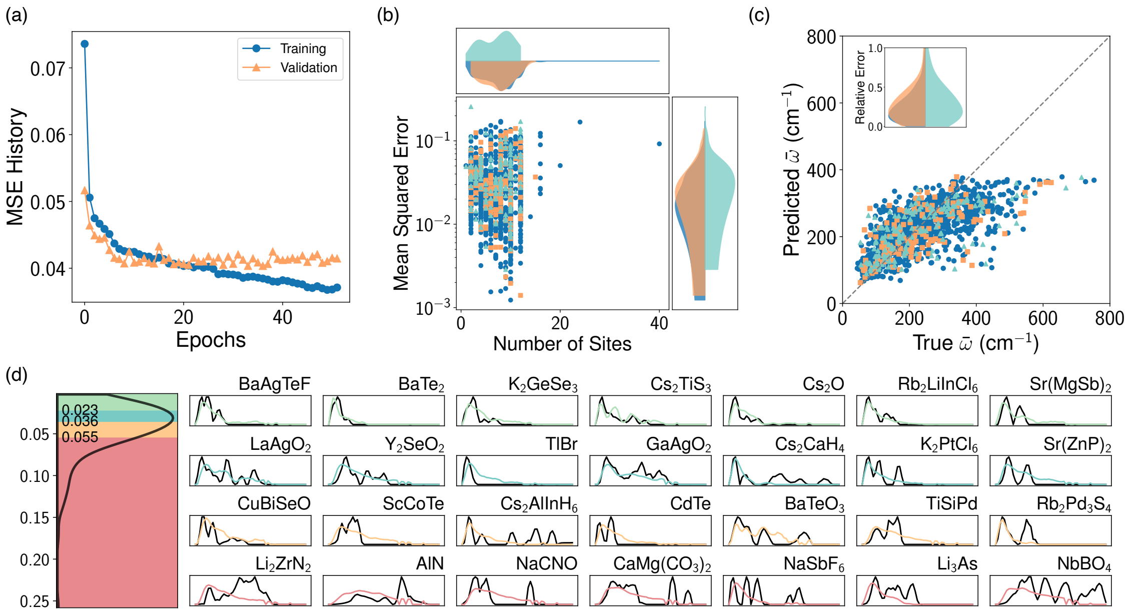

Figure S16. Phonon DoS predicted by the convolutional neural network.

COMPARISON TO CONVOLUTIONAL NEURAL NETWORK APPROACH

In order to better quantify the advantages of the tensor field neural network, we present a brief comparison to a convolutional neural network (CNN). Here, the unit cells are encoded as three-dimensional densities, similar to [58]. In particular, they are defined by

$$\rho_Z(\boldsymbol{r}) = \sum_{i=1}^{N} Z_i \mathrm{e}^{-|\boldsymbol{r}-\boldsymbol{r}_i|^2}, \qquad \rho_m(\boldsymbol{r}) = \sum_{i=1}^{N} m_i \mathrm{e}^{-|\boldsymbol{r}-\boldsymbol{r}_i|^2}, \tag{S6}$$

and mapped onto a discrete three-dimensional grid with positions of grid points determined by corresponding lattice vectors and parameters. The densities are then input into a three-dimensional convolutional neural network (architecture is shown in Table S3). Same training, validation, and test samples are used here. In particular, to

consider lattice periodicity, the densities for each unit cell are calculated in the middle of a $3 \times 3 \times 3$ super cell to account for contributions from neighbors. For each material, four unit cells with randomly translated atomic positions (shifted origins) alongside the original unit cell are used to train and validate the model, in order to achieve representation-independent outputs. The resulting performances are summarized in Figure S16 and are found not as good as E(3)NN's. Specifically, the CNN seems intended to predict similar profiles regardless of specific atomic structures, and unable to capture high energy peaks. Such performance further results in lower predicted average frequency, the failure in capturing main features makes it unsuitable to be applied on unseen materials.

Admittedly, there are several ways to improve the current CNN architecture. One noticeable problem is that the convolutional kernel here acts on different length scales depending on the lattice. A fixed grid like in [58] can also be adopted to resolve this issue, but other constraints such as limited representation ability (confined by grid dimension) and lowered spatial resolution will be brought up. In addition, more data augmentation will also be helpful. However, the E(3)NN offered a more physics-informed framework to intrinsically consider rotation and translation equivariance while get rid of data augmentation to capture arbitrary orientations and periodicity of the unit cell. In this case, the parameters in E(3)NN are more focused on building direct connections between crystal structures and target properties without interference from other information. As a result, it can achieve higher prediction accuracy and better generalization ability.
